# Digital Interventions in Neuro‐Rehabilitation: Gotcha! app‐based therapy for proper name anomia in people with dementia

**DOI:** 10.1002/alz.083779

**Published:** 2025-01-09

**Authors:** Aygun Badalova

**Affiliations:** ^1^ UCL, institute Of Neurology, London, United Kingdom United Kingdom

## Abstract

**Background:**

Proper name anomia is a common experience that can become amplified in patients with a diagnosis of dementia (PWD). The Gotcha! app aims to provide practice‐based therapy for PWD to relearn the names of key people in their lives. It has been developed using the principles of errorless learning and spaced retrieval, which have previously been shown to improve the remembering the familiar people’s names and benefit the relationship between the PWD and their loved ones. (Clare et al, 1999, 2000, 2003).

**Methods:**

Gotcha! is a digital confrontation naming therapy app which enables patients to train one face per day by using photos of the person whose name is to be trained. We employed a single‐case experimental design with weekly testing of free‐naming in both six‐week blocks (pre therapy and therapy). During the therapy, a novel speech verifier is used to provide real‐time feedback (Barbera et al. 2020). Two analyses method is used to investigate the behavioural data: 1) within‐subject non‐parametric analysis using Tau‐U metric (Parker et al. 2011); 2) a parametric group analysis using an ANOVA.

**Results:**

The trial is ongoing. Results from the first 20 subjects show:

1) Tau‐U. 80% showed a positive trend with better naming during the training phase with 8/20 reaching statistical significance.

2) ANOVA demonstrated a significant effect at the group level of training>baseline phase, F (1,19) = 13.18, p = 0.01

**Conclusion:**

App‐based proper name anomia retraining works for the majority of PWD in our trial thus far. Being able to freely recall and produce the name of a relative or loved one has a big impact on people’s lives.

